# Impact of Childhood Abuse on the Risk of Non-Suicidal Self-Injury in Mainland Chinese Adolescents

**DOI:** 10.1371/journal.pone.0131239

**Published:** 2015-06-26

**Authors:** Yuhui Wan, Jing Chen, Ying Sun, Fangbiao Tao

**Affiliations:** 1 Department of Maternal, Child & Adolescent Health, School of Public Health, Anhui Medical University, 81 Meishan Road, Hefei, 230032, Anhui, China; 2 Anhui Provincial Key Laboratory of Population Health & Aristogenics, 81 Meishan Road, Hefei, 230032, Anhui, China; University of Stellenbosch, SOUTH AFRICA

## Abstract

**Background:**

Childhood abuse has been associated with significant increases in non-suicidal self-injury (NSSI) behaviors in adolescents; however, only general definitions of this risk indicator have been examined. This study identified relationships between specific forms of childhood abuse and NSSI in mainland Chinese adolescents.

**Method:**

A total of 14,221 cases were retained from an epidemiological study involving adolescents from junior and senior middle schools. Information relating to the perpetrator, perceived harm, timing of exposure to different types of childhood abuse, and NSSI were obtained. Logistic regression was used to analyze relationships between each form of childhood abuse and NSSI.

**Results:**

Approximately 51.0% of the students reported at least one abusive childhood experience. Nearly one in four students (24.9%) reported that they had engaged in NSSI in the past 12 months. Each type of childhood abuse, occurring at any time within the first 16 years of life, especially in situations of continuous exposure, was significantly associated with NSSI. A significant graded relationship was found between number of abusive childhood experiences and NSSI. Students maltreated by parents or others were at high risk of engaging in NSSI, the risk was greater in students maltreated by both; students who had been exposed to childhood abuse with no perceived harm still demonstrated an elevated risk for NSSI. The pattern of associations did not vary by gender.

**Conclusions:**

These findings suggest that experiencing any of various forms of childhood abuse should be considered a risk factor for NSSI during adolescence. Further research should focus upon psychosocial, neural, and genetic factors that might moderate or mediate the onset of NSSI in adolescents who have experienced childhood abuse.

## Introduction

Non-suicidal self-injury (NSSI) is an act with a nonfatal outcome in which an individual deliberately initiates injurious behavior (such as self-cutting) or ingests a toxic substance or object with the intention of causing injury to themselves for purposes that are not culturally sanctioned [1,2]. NSSI is now recognized as a widespread and pervasive public health concern, occurring at significant rates within school-based samples of children and adolescents worldwide [3], and is one of the strongest predictors of completed suicide [4]. Substantial research has been conducted attempting to identify risk factors for NSSI with the aim of developing effective prevention and early intervention. A consistently cited risk factor is childhood abuse [5,6].

Several studies have retrospectively investigated various groups of adverse childhood experiences as risk factors for NSSI in adolescence or adulthood. For example, a Finnish study indicated that girls who had been exposed to childhood sexual abuse were at a significantly increased risk of NSSI [7]. In addition, deliberate self-harm has been associated with physical and emotional abuse in late adolescence [8] and Duke et al. have shown that exposure to any adverse childhood experience, regardless of the type of event, increased the risk of self-harm between 2.7 and 6.1 times [9]. Despite a growing interest in the role of childhood abuse in the development of NSSI later in life, the majority of studies have used only general definitions of this risk indicator. Such studies have not considered the effects of specific perpetrators or number of abusive experiences. Moreover, to date, no studies have investigated the role of the timing of childhood abuse in relation to NSSI. Thus, the relationships between childhood abuse and NSSI have been methodologically limited, while lack of detailed information on childhood abuse, including type, perpetrator, timing etc.

Previous research emphasizes the importance of accounting for these specific aspects of childhood abuse as they may vary with respect to their impact on subsequent mental health and risky behaviors. For instance, a large retrospective cohort study has indicated a greater risk of attempted suicide following the occurrence of multiple versus single or no adverse experiences [10]. Brown et al. posited that severe maltreatment perpetrated by the main maternal figure during childhood has a more central etiological role in adult women’s depression than abuse committed by a paternal figure; however, the timing of the maltreatment was unrelated to risk of chronic depression [11]. Another study suggested that individuals who experienced psychosis were three times more to report having exposed to severe maternally perpetrated physical abuse less than 12 years, but other forms of adversity, such as maternal neglect and sexual abuse and paternal maltreatment, were not associated with psychosis [12]. Furthermore, a study of an incarcerated population indicated that suicidal inmates had more sexual abuse, physical maltreatment, and emotional maltreatment experiences than non-suicidal inmates, and had experienced more traumatic life events during childhood, later life, and incarceration [13]. Consequently, before the research focus can be shifted to the mechanisms underlying the association between childhood abuse and NSSI, it is imperative to determine precisely which aspects of childhood abuse drive this association.

Therefore, a large-scale school-based survey was conducted to investigate the prevalence of childhood abuse and NSSI in adolescents in mainland China and evaluate the relationship between different forms of childhood abuse and risk of NSSI. We hypothesized that significant associations between each form of childhood abuse and adolescent NSSI would be found. Previous research findings [7.9] predicted that the pattern of associations would be different by gender. We especially hypothesized that identified pattern of group differences would vary between male and female.

## Methods

### Sample and Procedures

The study data were taken from an epidemiological study involving adolescents from junior and senior middle schools located in four provinces of China between November and December 2012. As China encompasses a vast territory with diverse geographic and economic development, we selected four sampled cities from four areas: southern (Guangzhou in Guangdong province), central (Xinxiang in Henan province), northern (Shenyang in Liaoning province), and western (Chongqing) areas. Collectively these cities represent the overall cultures of China, the economically developed region and the developing interior regions of the country. Eight schools (including four rural junior and senior schools and four urban junior and senior schools) were selected from each city, all of which were general junior and senior schools (excluding experimental or key schools). A total of 14,665 students from grades 7–12 were recruited to participate in the study. Four hundred fifty-four of the 14,665 sampled students were excluded from the study because of (1) absence from school on the day of the survey or unwilling to respond to the questionnaire, and (2) high levels of missing data or obviously fictitious or inconsistent responses. Thus, questionnaire data from 14,211 of the 14,665 sampled students were retained for analysis. The mean age of the participating students was 15.1 years (SD = 1.9) and 7,509 participants (52.8%) were female.

Written informed consent could not be implemented in such large sample population-based study in China. However, the study insured all the participants and their guardians are aware of the purpose and content of this investigation. During the organization period, we had signed informed consent with each participating school, including the principal of each selected classroom. One week prior to screening day, the parents or guardians of the students were informed of the study through a notice sent home from the schools asking them to contact the teachers by phone if they did not wish their child to participate in the survey. Prior to the formal investigation, the team members explained the anonymous and confidential nature of the data to the students, and provided an opportunity for them to ask questions. If they were not willing to participate, they were allowed to withdraw from the study. Each center used an anonymous questionnaire (S1 Questionnaire—Physical and psychological health for adolescents) for data collection. Completion of the self-reported questionnaire took approximately 25 minutes. A teacher was always present in the classroom but was not permitted to intervene in the research procedure. We reported the incidents of abuse and non-suicidal self-injury to each principal of selected school and the principal of each selected classroom. The feedback may be helpful for them to understand the whole picture of students' incidents of abuse and non-suicidal self-injury so as to accordingly take measures for intervention (eg. health education). Approval for the design and data collection procedures, including passive consent from parents, was obtained by the Ethics Committee of Anhui Medical University (2012534).

## Variables

### Childhood Abuse

Abusive childhood experiences were measured using a modified version of the Parent-Child Conflict Tactics Scale [14] and the Centers for Disease Control and Prevention Short ACE Tool [15]. All of the questions used to reveal abusive childhood experiences were introduced with the phrase, ‘‘While you were growing up (during your first 16 years of life), how often did someone do any of these things to you—very often, often, sometimes, occasionally, or never?”

Physical abuse was defined using 5 questions: Did a parent or other adults in the household ever (1) push, grab, pinch, or throw something at you seriously; (2) slap you on the arm, hand, leg, head, ears, or face seriously; (3) hit you with fist or kick you hard; (4) force you to kneel on the ground or stand for a long time; or (5) hit you on some part of your body with something like a belt, hairbrush, stick, or some other hard object?

Emotional abuse was defined using four questions: Did a parent or other adults in the household ever (1) send you away or kick you out of the house; (2) shout, yell, or scream at you in front of others; (3) swear or curse at you; or (4) call you dumb, lazy, or another name like that?

Sexual abuse was defined using four questions: Did an adult or person at least five years older ever (1) touch or fondle you in a sexual way; (2) have you touch their body in a sexual way; (3) attempted (or actually had) intercourse with you; (4) force you to watch pornographic pictures or videos or talk dirty to you?

Respondents were defined as exposed to a category if they responded “very often,” “often,” “sometimes,” or “occasionally” to any item in that category. Responses of “very often,” “often,” “sometimes,” or “occasionally” to any of the above 13 questions defined childhood abuse. Cronbach’s α coefficient for the physical, emotional, and sexual abuse subscales and the overall scale were 0.826, 0.784, 0.830, and 0.871 respectively.

Because of the high interrelatedness of various types of childhood abuse (all *P*< 0.01), an ordinal “number of different types of childhood abuse” score was created by summing the dichotomous childhood abuse items (range: 0 [unexposed] to 13 [exposed to all experiences]) to investigate the graded association between the number of different types of childhood abuse experienced and NSSI. Due to the relatively small sample sizes, childhood abuse scores of ≥ 7 were combined. Thus, analyses were conducted with 5 categories of summed score (0, 1–2, 3–4, 5–6, or ≥ 7), with 0 experiences as the referent.

For those who confirmed exposure to childhood abuse, details of the perpetrator of the abuse (parents, others) and perceived harm (no harm, mild, moderate, and severe) regarding the abuse were requested. In the data analysis, perpetrators of abuse categories were reassigned as maltreated by parents only, maltreated by others only, or maltreated by both; perceived harm categories were reassigned as abuse with no harm, mild harm, or moderate or severe harm.

Additionally, the timing of exposure to childhood abuse was investigated. In the data analysis, timing of exposure to abusive childhood experiences was grouped into early only (0y–9y only in junior middle school students and 0y–12y only in senior middle school students), late only (9y–presentation only in junior middle school students and 12y–16y only in senior middle school students), and continuous (0y–presentation in junior middle school students and 0y–16y in senior middle school students).

## NSSI

The questionnaire included a screening question for NSSI, which asked “Within the last year, have you harmed yourself in a way that was deliberate but not intended as a means by which to take your life? Yes or No.” [16,17]. A list of several NSSI methods (hitting, pulling hair, banging head, pinching, biting, cutting, overdosing, and ingesting non-ingestible substance,) was then presented. The details of the questions were as follows: (1) hit yourself? (2) pulled your own hair? (3) banged your head or fist against something? (4) pinched or scratched yourself? (5) bitten yourself? (6) cut or pierced yourself? (7) taken an overdose (e.g. of pills, alcohol or cigarette)? (8) ingested a non-ingestible substance or object? For those who confirmed that they had engaged in the behaviors were coded as NSSI. The internal consistency reliability of NSSI was 0.780 in the present study.

### Psychological Symptoms

Psychological symptoms were measured using the psychological domain of the Multidimensional Sub-health Questionnaire of Adolescents (MSQA), which consisted emotional symptoms; conduct symptoms and social adaptation symptoms. The psychological domain showed good internal consistency (Cronbach’s alpha = 0.957), test-retest reliability (Cohen’s k = 0.868), and split-half reliability coefficient (r = 0.942). Details of the instrument were documented elsewhere [18].

### Control Variables

Demographic characteristics and conditions known or thought to be correlates of NSSI were measured [16,19], including gender (boys or girls), age (≤15y or >15y), registered residence (urban or rural), only child (yes or no), parents’ education level (less than junior middle school, junior middle school, senior middle school, college or more), perceived family economic status (poor, moderate, or good), and numbers of friends (0, 1–2, 3–5, ≥6).

### Statistical Analysis

In this study, a chi-square test was performed to examine gender differences in each form of childhood abuse and NSSI. The differences in potential covariates (such as age, parents’ education, perceived family economical status, number of friends and psychological symptoms) between the participants who did and did not engage in NSSI were also evaluated using a chi-square test. Logistic regression was used to analyze relationships between each form of childhood abuse and NSSI according to gender. In the final logistic regression models, potential covariates were controlled for. All analyses were conducted with SPSS software, version 10 (SPSS Inc., Chicago, IL). Given the size of the sample, a P-value of <0.01 was considered statistically significant in the analyses.

## Results

### Distribution of Childhood Abuse and NSSI by Gender

Table 1 shows the prevalence of each type of childhood abuse by gender. More than half of the students (51.0%) reported at least one abusive childhood experience. Girls had significantly greater exposure to emotional abuse (*P*<0.001), and boys had significantly greater exposure to physical abuse (*P*<0.001), while no gender differences were found in exposure to sexual abuse and overall childhood abuse (*P*>0.01).

**Table 1:** Distribution of childhood abuse by gender, n(%).

Variable	Total	Boys	Girls	*χ* ^*2*^-value	*p*-value
Type of abuse					
Physical abuse	5824(41.0)	2906(43.3)	2918(38.9)	28.835	<0.001
Emotional abuse	5409(38.0)	2436(36.3)	2973(39.6)	16.369	<0.001
Sexual abuse	1039(7.3)	502(7.5)	537(7.2)	0.562	0.453
Childhood abuse	7246(51.0)	3450(51.4)	3796(50.6)	1.019	0.313
Number of childhood abuse				20.841	<0.001
0	6975(49.0)	3262(48.6)	3713(49.4)		
1~2	3137(22.1)	1433(21.3)	1704(22.7)		
3~4	2025(14.2)	968(14.4)	1057(14.1)		
5~6	1140(8.0)	540(8.0)	600(8.0)		
≥7	944(6.6)	509(7.6)	435(5.8)		
Perpetrator of childhood abuse				60.380	<0.001
Parents only	1860(13.1)	777(11.6)	1083(14.4)		
Others only	1747(12.3)	953(14.2)	794(10.6)		
Both	3639(25.6)	1720(25.6)	1919(25.6)		
Perceived harm of childhood abuse				8.030	0.018
Mild	3578(25.2)	1648(24.6)	1930(25.7)		
Moderate and severe	2863(20.1)	1416(21.1)	1447(19.3)		
Timing of childhood abuse				12.591	0.006
Early only	1984(14.0)	900(13.4)	1084(14.4)		
Late only	2198(15.5)	1109(16.5)	1089(14.5)		
Continuous	3064(21.5)	1441(21.5)	1623(21.6)		

Table 2 shows the prevalence of each form of NSSI behaviors by gender. A total of 3,546 (24.9%) students reported that they had engaged in NSSI during the previous 12 months. Some forms of NSSI, such as bumping head and overdosing were reported significantly higher among boys than girls (*P*<0.001). While pinching, biting, and cutting were higher among girls than boys (*P*<0.001). The rate of hitting, pulling hair, ingesting and total NSSI behaviors revealed no statistically significant differences by gender (P >0.01).

**Table 2:** Forms of NSSI behaviors by gender, n(%).

Gender	Hitting	Pulling hair	Bumping head	Pinching	Biting	Cutting	Overdosing	Ingesting	Total*
Boys	799(11.9)	629(9.4)	1281(19.1)	405(6.0)	262(3.9)	228(3.4)	218(3.2)	61(0.9)	1734(25.8)
Girls	861(11.5)	612(8.2)	874(11.6)	850(11.3)	509(6.8)	368(4.9)	152(2.0)	44(0.6)	1812(24.1)
Total	1660(11.7)	1241(8.7)	2156(15.2)	1255(8.8)	771(5.4)	596(4.2)	370(2.6)	105(0.7)	3546(24.9)

* anyone of NSSI

### Associations between Socio-Demographic Characteristics, Psychological Symptoms, Childhood Abuse, and NSSI

Considering each explanatory variable separately, we found that age, mother’s education level, perceived family economic status, numbers of friends and psychological symptoms were significantly associated with each type of childhood abuse (*P*<0.01). Father’s education level was significantly associated with physical abuse, sexual abuse, and overall childhood abuse (*P*<0.01), and being an only child was significantly associated with overall childhood abuse (*P*<0.01). Additionally, age, father’s education level, perceived family economic status, numbers of friends and psychological symptoms were significantly associated with NSSI (*P*<0.01).

### Bivariate and Multivariate Associations between Type, Number, Perpetrator, Perceived Harm, and Timing Of Childhood Abuse and NSSI by Gender

Table 3 shows that the students’ exposure to childhood abuse, regardless of type, demonstrated a comparably high rate of risk (approximately 2.5–4 times higher) of NSSI. When the 3 types of abuse were entered simultaneously, physical abuse (Boys, 2.17(1.89–2.50); Girls, 2.16(1.89–2.48)), emotional abuse (Boys, 2.17(1.88–2.49); Girls, 1.90(1.66–2.18)) and sexual abuse (Boys, 1.40(1.14–1.71); Girls, 1.76(1.45–2.14)) remained significant for boys and girls (*P*<0.01).

**Table 3:** Bivariate and multivariate associations between type, number, perpetrator, perceived harm and timing of childhood abuse and NSSI by gender, OR(95%CI).

Variable	Boys			Girls		
	No. (%)	Model 1^a^	Model 2^a^ ^,^ ^b^	No. (%)	Model 1^a^	Model 2^a^ ^,^ ^b^
Type						
Physical abuse						
No	565(14.8)	1.0	1.0	666(14.5)	1.0	1.0
Yes	1169(40.2)	3.86(3.44–4.34^)^ ^#^	3.35(2.96–3.78^)^ ^#^	1146(39.3)	3.81(3.44–4.34)^#^	3.21(2.86–3.60)^#^
Emotional abuse						
No	684(16.0)	1.0	1.0	663(14.6)	1.0	1.0
Yes	1050(43.1)	3.98(3.55–4.46^)^ ^#^	3.39(3.01–3.81^)^ ^#^	1149(38.6)	3.68 (3.30–4.11)^#^	3.00(2.67–3.36)^#^
Sexual abuse						
No	1480(23.8)	1.0	1.0	1540(22.1)	1.0	1.0
Yes	254(50.6)	3.27(2.72–3.94^)^ ^#^	2.50(2.06–3.03^)^ ^#^	272(50.7)	3.62(3.03–4.33)^#^	2.81(2.33–3.40)^#^
Childhood abuse						
No	402(12.3)	1.0	1.0	423(11.4)	1.0	1.0
Yes	1332(38.6)	4.47(3.95–5.07^)^ ^#^	3.88(3.41–4.41)	1389(36.6)	4.49(3.98–5.07)^#^	3.73(3.29–4.22)^#^
Number of childhood abuse						
0	402(12.3)	1.0	1.0	423(11.4)	1.0	1.0
1~2	398(27.8)	2.74(2.34–3.20)^#^	2.62(2.24–3.07)^#^	450(26.4)	2.79(2.41–3.24)^#^	2.58(2.22–3.00)^#^
3~4	370(38.2)	4.40(3.73–5.20)^#^	3.90(3.29–4.62)^#^	411(38.9)	4.95(4.22–5.81)^#^	4.20(3.57–4.96)^#^
5~6	276(51.1)	7.44(6.10–9.07)^#^	6.20(5.06–7.60)^#^	281(46.8)	6.85(5.67–8.28)^#^	5.18(4.25–6.31)^#^
≥7	288(56.6)	9.27(7.56–11.37)^#^	6.84(5.53–8.45)^#^	247(56.8)	10.22(8.24–12.67)^#^	7.09(5.66–8.89)^#^
Perpetrator						
Physical abuse						
No abuse	565(14.8)	1.0	1.0	666(14.5)	1.0	1.0
Parents only	285(33.6)	2.91(2.46–3.44)^#^	2.75(2.31–3.26)^#^	432(35.9)	3.29(2.85–3.80)^#^	2.92(2.52–3.39)^#^
Others only	322(36.6)	3.31(2.81–3.90)^#^	2.90(2.45–3.44)^#^	216(36.5)	3.38(2.81–4.08)^#^	2.85(2.34–3.47)^#^
Both	562(47.7)	5.23(4.52–6.04)^#^	4.26(3.69–4.98)^#^	496(44.2)	4.66(4.03–5.37)^#^	3.70(3.18–4.29)^#^
Emotional abuse						
No abuse	684(16.0)	1.0	1.0	663(14.6)	1.0	1.0
Parents only	264(40.1)	3.51(2.94–4.19)^#^	3.22(2.69–3.86)^#^	371(35.1)	3.16(2.72–3.67)^#^	2.71(2.32–3.17)^#^
Others only	354(39.8)	3.47(2.96–4.06)^#^	2.98(2.54–3.50)^#^	312(35.8)	3.26(2.78–3.83)^#^	2.69(2.27–3.18)^#^
Both	432(48.7)	4.99(4.27–5.82)^#^	4.00(3.41–4.70)^#^	466(44.6)	4.70(4.06–5.45)^#^	3.62(3.10–4.22)^#^
Sexual abuse						
No abuse	1480(23.8)	1.0	1.0	1540(22.1)	1.0	1.0
Parents only	6(46.2)	2.74(0.92–8.16)	1.61(0.51–5.10)	7(41.2)	2.47(0.94–6.50)	1.55(0.56–4.28)
Others only	228(49.7)	3.15(2.60–3.82)^#^	2.43(1.98–2.97)^#^	256(51.9)	3.81(3.17–4.59)^#^	2.98(2.45–3.64)^#^
Both	20(66.7)	6.39(2.99–13.69)^#^	4.71(2.14–10.36)^#^	9(33.3)	1.76(0.79–3.93)	1.37(0.59–3.19)
Childhood abuse						
No abuse	402(12.3)	1.0	1.0	423(11.4)	1.0	1.0
Parents only	236(30.4)	3.10(2.58–3.74)^#^	2.97(2.46–3.59)^#^	331(30.6)	3.42(2.91–4.03)^#^	3.10(2.62–3.67)^#^
Others only	308(32.3)	3.40(2.86–4.03)^#^	3.05(2.56–3.63)^#^	244(30.7)	3.45(2.88–4.14)^#^	2.97(2.46–3.59)^#^
Both	788(45.8)	6.02(5.22–6.93)^#^	4.95(4.29–5.73)^#^	814(42.4)	5.73(5.00–6.56)^#^	4.51(3.92–5.20)^#^
Perceived harm						
Physical abuse						
No abuse	565(14.8)	1.0	1.0	666(14.5)	1.0	1.0
No harm	125(32.1)	2.71(2.15–3.41)^#^	2.56(2.02–3.24)^#^	123(28.0)	2.29(1.82–2.86)^#^	2.00(1.59–2.52)^#^
Mild	566(36.4)	3.29(2.87–3.77)^#^	3.02(2.62–3.47)^#^	630(37.7)	3.57(3.14–4.06)^#^	3.23(2.83–3.69)^#^
Moderate and severe	478(49.7)	5.67(4.85–6.61)^#^	4.44(3.78–5.22)^#^	393(48.6)	5.57(4.74–6.54)^#^	4.00(3.38–4.74)^#^
Emotional abuse						
No abuse	684(16.0)	1.0	1.0	663(14.6)	1.0	1.0
No harm	124(34.8)	2.81(2.22–3.54)^#^	2.63(2.07–3.33^)#^	90(24.1)	1.86(1.45–2.39)^#^	1.65(1.27–2.13)^#^
Mild	449(39.0)	3.36(2.91–3.88)^#^	3.08(2.66–3.57)^#^	531(35.5)	3.21(2.81–3.67)^#^	2.85(2.48–3.28)^#^
Moderate and severe	477(51.3)	5.54(4.76–6.46)^#^	4.23(3.60–5.00)^#^	528(47.9)	5.36(4.65–6.20)^#^	3.86(3.31–4.49)^#^
Sexual abuse						
No abuse	1480(23.8)	1.0	1.0	1540(22.1)	1.0	1.0
No harm	54(47.8)	2.93(2.01–4.25)^#^	2.73(1.85–4.01)^#^	40(46.0)	3.00(1.96–4.59)^#^	2.78(1.77–4.35)^#^
Mild	91(48.4)	3.00(2.24–4.02)^#^	2.45(1.81–3.33)^#^	137(51.1)	3.69(2.88–4.18)^#^	3.02(2.33–3.93)^#^
Moderate and severe	109(54.2)	3.79(2.85–5.03)^#^	2.41(1.79–3.26)^#^	95(52.2)	3.85(2.86–5.18)^#^	2.54(1.85–3.48^)#^
Childhood abuse						
No abuse	402(12.3)	1.0	1.0	423(11.4)	1.0	1.0
No harm	111(28.8)	2.87(2.25–3.67)^#^	2.78(2.17–3.56)^#^	94(22.4)	2.25(1.75–2.89)^#^	2.07(1.60–2.68)^#^
Mild	546(33.1)	3.53(3.05–4.08)^#^	3.33(2.87–3.86)^#^	616(31.9)	3.65(3.17–4.19)^#^	3.34(2.90–3.85)^#^
Moderate and severe	675(47.7)	6.48(5.59–7.51)^#^	5.08(4.36–5.92)^#^	679(46.9)	6.88(5.95–7.95)^#^	5.04(4.33–5.87)^#^
Timing						
Physical abuse						
No abuse	565(14.8)	1.0	1.0	666(14.5)	1.0	1.0
Early only	367(34.5)	3.02(2.59–3.53)^#^	2.66(2.27–3.11)^#^	454(34.1)	3.05(2.65–3.51)^#^	2.66(2.30–3.07)^#^
Late only	361(37.5)	3.45(2.94–4.04)^#^	3.08(2.62–3.63)^#^	303(39.4)	3.83(3.25–4.53)^#^	3.34(2.81–3.97)^#^
Continuous	441(50.1)	5.76(4.91–6.76)^#^	4.80(4.08–5.66)^#^	389(47.6)	5.36(4.56–6.29)^#^	4.13(3.49–4.88)^#^
Emotional abuse						
No abuse	684(16.0)	1.0	1.0	663(14.6)	1.0	1.0
Early only	257(38.2)	3.25(2.73–3.88)^#^	2.85(2.38–3.41)^#^	283(31.2)	2.65(2.26–3.12^)#^	2.31(1.95–2.73)^#^
Late only	436(40.3)	3.54(3.06–4.10)^#^	3.11(2.68–3.62)^#^	490(39.3)	3.78(3.29–4.35)^#^	3.16(2.73–3.65)^#^
Continuous	357(52.4)	5.79(4.88–6.87)^#^	4.61(3.86–5.50)^#^	376(45.9)	4.95(4.22–5.81)^#^	3.65(3.09–4.32)^#^
Sexual abuse						
No abuse	1480(23.8)	1.0	1.0	1540(22.1)	1.0	1.0
Early only	46(46.9)	2.83(1.89–4.22)^#^	2.17(1.43–3.31)^#^	65(48.1)	3.28(2.33–4.61)^#^	2.46(1.71–3.54)^#^
Late only	161(50.9)	3.32(2.64–4.17)^#^	2.59(2.04–3.29)^#^	176(51.5)	3.74(3.00–4.66)^#^	3.00(2.38–3.78)^#^
Continuous	47(53.4)	3.66(2.40–5.59)^#^	2.54(1.63–4.00)^#^	31(51.7)	3.77(2.27–6.28)^#^	2.63(1.53–4.51)^#^
Childhood abuse						
No abuse	402(12.3)	1.0	1.0	423(11.4)	1.0	1.0
Early only	277(30.8)	3.16(2.65–3.77)^#^	2.89(2.42–3.46)^#^	298(27.5)	2.95(2.49–3.49)^#^	2.65(2.23–3.15)^#^
Late only	368(33.2)	3.53(3.00–4.16)^#^	3.20(2.71–3.78)^#^	379(34.8)	4.15(3.54–4.88)^#^	3.56(3.01–4.20)^#^
Continuous	687(47.7)	6.48(5.62–7.51)^#^	5.33(4.58–6.20)^#^	712(43.9)	6.08(5.28–7.00)^#^	4.76(4.11–5.51)^#^

^a^ OR (unadjusted), calculated using weighted data.

^b^ Adjusted for age, father’s education, perceived family economical status, number of friends and psychological symptoms.

^#^ *P*<0.01

In adjusted models, a significant graded relationship was found between the number of abusive childhood events and NSSI. There was also evidence that students maltreated by either parents or others were at high risk of engaging in NSSI, this risk was greater in students maltreated by both. When maltreated by parents was used as the reference category, maltreated by both (Boys: Physical abuse 1.56(1.29–1.88), Childhood abuse 1.67(1.39–2.01); Girls: Physical abuse 1.27(1.06–1.51), Emotional abuse 1.34(1.11–1.61), Childhood abuse 1.46(1.24–1.72)) showed a comparably high rate of risk of NSSI (*P*<0.01).

Furthermore, students who were exposed to childhood abuse with no perceived harm demonstrated an elevated risk for NSSI similar to those with perceived mild, moderate and severe harm. With regard to the timing of childhood abuse, exposure to any type of abuse during any period within the first 16 years of life, especially in situations of continuous exposure, was significantly associated with NSSI. When abused early only was used as the reference category, abused continuously (Boys: Physical abuse 1.81(1.50–2.18), Emotional abuse 1.62(1.29–2.02), Childhood abuse 1.84(1.54–2.21); Girls: Physical abuse 1.55(1.29–1.87), Emotional abuse 1.58(1.29–1.94), Childhood abuse 1.79(1.51–2.13)) revealed a higher rate of risk of NSSI (*P*<0.01).

## Discussion

### Prevalence of Childhood Abuse and NSSI

Findings from this sample show that approximately 51.0% of the students reported at least one abusive childhood experience. The prevalence rates of childhood physical, emotional, and sexual abuse in the current study (41.0%, 38.0% and 7.3% respectively) are lower than in previous studies concerning Chinese populations and one global study [20,21,22], but much higher than in findings from Western countries and some other studies conducted in China [23,24,25]. Findings regarding gender differences in childhood abuse have also been mixed. The current study found a higher rate of physical abuse in boys and a higher rate of emotional abuse in girls, while no gender difference was found with respect to sexual abuse. In contrast, a study conducted with students in Guangzhou demonstrated that significantly more boys than girls experienced very severe physical and sexual abuse, while there was no gender difference in exposure to psychological aggression [21]. A study from Finland indicated that girls had significantly greater exposure to sexual abuse, while no difference was found with respect to exposure to physical abuse by gender [7]. Such differences highlight the need to re-examine the definition of childhood abuse. It has been suggested that cultural influence should also be considered when interpreting the results of any child maltreatment research [21,26,27].

The 24.9% prevalence rate of NSSI reported in this study is similar to rates reported in previous studies [28,29], higher than in studies conducted with Irish and Japanese adolescents [19,30], and lower than in other studies with Australian and Hong Kong adolescents [31,32]. Our results showing that total NSSI behaviors revealed no statistically significant differences by gender are consistent with existing findings [33,34]; however, other findings regarding gender differences in NSSI have been mixed. For example, some studies have found that girls are more likely to engage in NSSI than boys [30,32], and other studies suggest that boys had significantly greater exposure than girls [35,36]. The definition of NSSI could be an important reason for the mixed findings. The lack of gender difference in this study may also reflect an actual lack of gender difference in rates across types of NSSI. For instance, a study with Chinese adolescents suggested that many forms of deliberate self-harm, such as pinching, grabbing, biting, and cutting, were reported to be significantly higher in girls, while hitting, hair pulling, and head banging were reported to be higher in boys; however, the total rate of deliberate self-harm behavior did not differ significantly according to gender [16].

### Specific Types of Childhood Abuse Associated with NSSI

Each type of childhood abuse was associated with a significantly increased risk of NSSI in this sample. This is inconsistent with findings from a study in which no relationship was found between childhood abuse and NSSI [37]. Previous findings regarding associations between specific types of childhood abuse and NSSI have also been contradictory. Glassman et al. reported that emotional abuse and sexual abuse were significantly associated with NSSI, while physical abuse had a nonsignificant relationship with NSSI [38]. However, a study with an undergraduate sample indicated that self-harm was not associated with a history of physical or sexual abuse, although many self-harm behaviors were associated with a history of emotional abuse [39]. Moreover, deliberate self-harm has been found to be associated with physical and emotional abuse in late adolescence [8]. In addition to differences in samples, the lack of consistency in variable definitions and analysis methods may account for the differing conclusions. In addition, significant results should be combined for effect size in the current study as even small differences may become significant in a large sample. Expanding upon these results, analyses in our findings demonstrated that we should also focus on those who have been exposed to childhood abuse with no perceived harm, because they reported significantly more NSSI than students who had not been exposed to childhood abuse. This suggests that the objective experience of exposure to childhood abuse led to a greater risk of NSSI than perceived harm.

In contrast to prior research, no significantly gender differences emerged when the risk factors for NSSI were examined separately for boys and girls. Swannell et al. found that varying types of child maltreatment predict NSSI differently, especially among females [40]. A study of adolescents in Finland also revealed that exposure to sexual abuse significantly increased the risk of NSSI in girls, but not boys [7]. However, the results of our study are consistent with those of a recent study conducted in the United States (US) that reported that childhood physical and sexual abuse were significantly associated with adolescent self-harm in boys and girls [9]. Moreover, measures of childhood maltreatment, sample size, gender proportion of sample etc. may be accounting for gender difference [7,40]. Future research should also focus on the inter-disciplinary and cross-cultured study to clarify and explain the relationship.

### Number of Childhood Abuse Experiences associated with NSSI

This study provided evidence of a dose-response effect for each type of childhood abuse on development of NSSI in adolescents. It is difficult to draw definitive conclusions from the existing research as formal tests of dose-response relationships between childhood abuse and NSSI have rarely been conducted [7]. Moreover, the current finding is consistent with previous studies that have demonstrated a cumulative effect of childhood adversity on risk of later health outcomes. For instance, a study of adults in the US indicated that there was a 30% to 40% increase in the risk of drug problems as abusive childhood adversity experience scores increased [41]. Mark et al. also found that higher childhood adversity experience scores increased the likelihood of smoking, heavy drinking, and morbid obesity in a dose-response manner [42]. However, Clausen and Crittenden suggested that single instances of certain types of abuse (e.g., physical or sexual) may be traumatic enough to produce detrimental effects, while other adverse experiences may require repeated exposure to cause harm to the child [43]. Unfortunately, we cannot test this hypothesis as the types of childhood adversity examined in this study were limited.

### Associations between Perpetrator of Childhood Abuse and NSSI

In our study, adolescents maltreated by either parents or others were at high risk of engaging in NSSI (except for parents-perpetrated sexual abuse) and the risk was greater in students maltreated by both. This is similar to the results of a recent study suggesting that childhood physical abuse by a household adult or sexual abuse by a family member increased the likelihood of self-harm [9]. However, this is inconsistent with previous studies demonstrating different risks for later health outcomes according to specific perpetrators of childhood abuse. For instance, Brown et al. found that maternal emotional abuse and paternal physical abuse, but not maternal physical abuse, increased the risk of later chronic depression in adult women [11]. A recent study indicated that sexual abuse perpetrated by a parental or parent figure (relative to another perpetrator) increased the incidence of multiple suicide attempts 12.27 times [44]. Another study found that sexual abuse committed by someone outside the family increased the level of dissociation more strongly than committed by family members [45]. However, to our knowledge few studies have investigated differential effects of the perpetrator of childhood abuse on NSSI; therefore, these comparisons are limited. Moreover, our data also revealed that maltreatment by parents and others during childhood led to a greater risk of NSSI, reflecting an accumulative effect of additional perpetrators.

### Associations between Timing of Exposure to Childhood Abuse and NSSI

Several studies have elucidated the effects of the timing of childhood adversities on the developmental course of mental health. Zink et al. found that age at first abuse had a linear dependence with trauma score decreasing by about half a point for each year of age [46]. Moreover, parents with a history (younger than 13 years for fathers and elder than 13 years for mothers) of childhood physical abuse showed a higher risk of becoming perpetrators of physical abuse with their children than parents without physical abuse experiences [47]. The results of the current study revealed a significant relationship between childhood abuse and NSSI across all child developmental stages. This supported the notion that vulnerability persists in children with early maltreatment, even if they are taken out of the problematic environment later and are exposed to a more beneficial environment subsequently [48]. Our data also suggested that consideration of factors other than the early onset of maltreatment is important in understanding the developmental pathways of NSSI. Additionally, continuous exposure to childhood abuse had the strongest association with NSSI, reflecting an accumulative effect of abusive events. These findings are consistent with theoretical models of reasons for engaging in NSSI [6].

### Limitations

The current study was a representative nationwide epidemiologic study of Chinese adolescents; moreover, it was one of the few published efforts to identify relationships between more specific forms of childhood abuse and NSSI in adolescents. However, several limitations should be considered when interpreting these results. First, the study began with adolescents in traditional school environments; as such, findings did not represent adolescents who were absent from school, which is important because studies have shown that self-injury and childhood abuse are more prevalent in individuals with lower educational achievement and socioeconomic status [8,49,50]. The extent to which one can generalize these findings to adolescents in other countries or cultures is also unclear as all participants in this study were adolescents from mainland China. Second, the data used in our study were cross-sectional, retrospective, and self-reported. Thus, the findings are correlational, and no determinations can be made about causal relationships between the variables examined in this study and memory bias cannot be avoided, although acceptable stability in reports of maltreatment and some other adverse childhood experiences has been found [51]. Third, the types of adversity examined were limited due to the formatting limitations of a school-based questionnaire designed to be administered during a class period, further investigation involving multiple adverse experiences should be conducted in future studies. Fourth, the current study involved some very young children (e.g., 10–11 years of age). Although efforts were made to assist these children in providing accurate responses to the survey questions, future studies should confirm that these children fully understand the questions in the survey.

## Conclusion

In conclusion, NSSI in adolescence is a relatively specific outcome of childhood abuse that is not dependent upon particular types of abuse. This indicated that effective childhood abuse prevention programs should address the vulnerabilities of the population and increase children and parents’ self-protection awareness and ability. Interventions that address NSSI may benefit from increased sensitivity to and recognition of a wide range of abusive childhood experiences. Further research should focus upon psychosocial, neural, and genetic factors that might moderate or mediate the onset of NSSI in adolescents who have experienced childhood abuse.

## Supporting Information

S1 Questionnaire Senior middle school Version. (DOC)
