# Supplementary material for: Impact of Childhood Abuse on the Risk of Non-Suicidal Self-Injury in Mainland Chinese Adolescents
Source: PLoS One. 2015 Jun 26;10(6):e0131239. doi: 10.1371/journal.pone.0131239 (PMC4482708; doi:10.1371/journal.pone.0131239)
Supplement: S1 Questionnaire — (DOC) [file pone.0131239.s001.doc]

**S1 Questionnaire - Physical and psychological health for adolescents**

**青少年身心健康问卷（高中版）**

1. **一般情况**

| 1. 性别 ① 男； ② 女 |
| --- |
| 2. 年级 ① 高一； ② 高二； ③ 高三 |
| 3. 出生日期 年 月 日 |
| 4. 户口所在地 ① 农村； ② 城镇 |
| 5. 你是否为独生子女 ① 是； ② 否 |
| 6. 与你一起生活时间最长的父亲（包括养父、继父）的文化程度 |
| ① 没有父亲； ② 父亲小学未毕业或没有上过学； ③ 小学毕业； |
| ④ 初中； ⑤ 高中或中专； ⑥ 大专或大专以上 |
| 7. 与你一起生活时间最长的母亲（包括养母、继母）的文化程度 |
| ① 没有母亲； ② 母亲小学未毕业或没有上过学； ③ 小学毕业； |
| ④ 初中； ⑤ 高中或中专； ⑥ 大专或大专以上 |
| 8. 你认为你的家庭经济条件与其他同学相比属于 |
| ① 差； ② 较差； ③ 中等； ④ 较好； ⑤ 好 |
| 9. 你有多少关系密切，可以得到支持和帮助的朋友 |
| ① 一个也没有； ② 1～2个； ③ 3～5个； ④ 6个或6个以上 |
| **二、行为情况** |
| 1. 最近1年内，你认真考虑过自杀吗？（即认真想过以自杀结束自己的生命） |
| 1. 没有； ② 考虑过1次； ③ 2～3次； ④ 4次以上 |
| 2. 最近1年内，你制定过怎样实施自杀的计划吗？（即认真想过或写过自杀的过程）  ① 没有； ② 考虑过1次； ③ 2～3次； ④ 4次以上 |
| 3. 最近1年内，你有过自杀行为吗？（如上吊、吃安眠葯、割手腕等） |
| 1. 没有； ② 考虑过1次； ③ 2～3次； ④ 4次以上 |

**三、情绪状况**

以下表格中列出了有些人可能会有的身体不适症状，请仔细阅读每一条，然后根据**最近一段时间**自己的实际感受，选择最符合你的一种情况，在后面相应的选项中划钩（**√**）。

| **题 目** | | **持续3个月以上** | **持续**  **2个月以上** | **持续**  **1个月以上** | **持续**  **2星期以上** | **持续**  **1星期以上** | **没有或持续不到1星期** |
| --- | --- | --- | --- | --- | --- | --- | --- |
|  | 对事物总是不感兴趣 | **1** | **2** | **3** | **4** | **5** | **6** |
|  | 总是很难记住学习内容 | **1** | **2** | **3** | **4** | **5** | **6** |
|  | 常常无缘无故地感到坐立不安 | **1** | **2** | **3** | **4** | **5** | **6** |
|  | 常常感到人们对我不友好 | **1** | **2** | **3** | **4** | **5** | **6** |
|  | 常常无缘无故地感到紧张 | **1** | **2** | **3** | **4** | **5** | **6** |
|  | | | | | | | |
|  | 总是感觉旁人能知道我的私下想法 | **1** | **2** | **3** | **4** | **5** | **6** |
|  | 经常责怪自己 | **1** | **2** | **3** | **4** | **5** | **6** |
|  | 常常感到有人在谈论我 | **1** | **2** | **3** | **4** | **5** | **6** |
|  | 常常害怕空旷的场所 | **1** | **2** | **3** | **4** | **5** | **6** |
|  | 在家里几乎很难安心学习 | **1** | **2** | **3** | **4** | **5** | **6** |
|  | | | | | | | |
|  | 做事经常犹豫不决 | **1** | **2** | **3** | **4** | **5** | **6** |
|  | 总是感到大多数人都不可信任 | **1** | **2** | **3** | **4** | **5** | **6** |
|  | 常常害怕去公共场合 | **1** | **2** | **3** | **4** | **5** | **6** |
|  | 对现在的学校生活常常感到不适应 | **1** | **2** | **3** | **4** | **5** | **6** |
|  | 常常无缘无故地感到心里烦躁 | **1** | **2** | **3** | **4** | **5** | **6** |
|  | | | | | | | |
|  | 当心情低落时，常常不愿向其他人倾诉 | **1** | **2** | **3** | **4** | **5** | **6** |
|  | 经常会无缘无故地感到害怕 | **1** | **2** | **3** | **4** | **5** | **6** |
|  | 总是感到前途没有希望 | **1** | **2** | **3** | **4** | **5** | **6** |
|  | 注意力无法集中 | **1** | **2** | **3** | **4** | **5** | **6** |
|  | 总是很难适应老师的教学方法 | **1** | **2** | **3** | **4** | **5** | **6** |
|  | | | | | | | |
|  | 常常感到苦闷 | **1** | **2** | **3** | **4** | **5** | **6** |
|  | 经常有想摔东西的冲动 | **1** | **2** | **3** | **4** | **5** | **6** |
|  | 头脑中总是有不必要的想法或字句盘旋 | **1** | **2** | **3** | **4** | **5** | **6** |
|  | 当遇到困难时，大多不想去求助于别人 | **1** | **2** | **3** | **4** | **5** | **6** |
|  | 上课时总是担心老师提问自己 | **1** | **2** | **3** | **4** | **5** | **6** |
|  | 总是不喜欢和同学、朋友在一起谈论问题 | **1** | **2** | **3** | **4** | **5** | **6** |
|  | 反复想到死 | **1** | **2** | **3** | **4** | **5** | **6** |
|  | 一天到晚对什么都提不起精神 | **1** | **2** | **3** | **4** | **5** | **6** |
|  | 经常想到怎样去实施自杀（如怎样去服毒、割腕、跳楼等） | **1** | **2** | **3** | **4** | **5** | **6** |
|  | 经常与人争论，非得争个胜负 | **1** | **2** | **3** | **4** | **5** | **6** |
|  | | | | | | | |
|  | 在人多的地方感到不自在 | **1** | **2** | **3** | **4** | **5** | **6** |
|  | 总觉得别人在跟我作对 | **1** | **2** | **3** | **4** | **5** | **6** |
|  | 单独一个人时总是感觉精神很紧张 | **1** | **2** | **3** | **4** | **5** | **6** |
|  | 总是很讨厌上学 | **1** | **2** | **3** | **4** | **5** | **6** |
|  | 经常不能控制地大发脾气 | **1** | **2** | **3** | **4** | **5** | **6** |
|  | | | | | | | |
|  | 一听说要考试，总感到坐立不安 | **1** | **2** | **3** | **4** | **5** | **6** |
|  | 经常因一些小事而愤怒 | **1** | **2** | **3** | **4** | **5** | **6** |
|  | 与同学相比，常常感到学习很困难 | **1** | **2** | **3** | **4** | **5** | **6** |
|  | 当别人看着我时，常常感到十分紧张 | **1** | **2** | **3** | **4** | **5** | **6** |

**四、最近1年内，你有没有以下故意伤害自己的行为？（不以自杀为目的）**

| **行 为** | **发生情况** |
| --- | --- |
| 1. 故意打自己 | ①有 次；　　②没有 |
| 2. 故意拽头发 | ①有 次；　　②没有 |
| 3. 故意撞头或用拳头击打其他物体 | ①有 次；　　②没有 |
| 4. 故意掐自己或抓伤自己 | ①有 次；　　②没有 |
| 5. 故意咬伤自己 | ①有 次；　　②没有 |
|  |  |
| 6. 故意割伤或刺伤自己 | ①有 次；　　②没有 |
| 7. 故意过量服用药物、饮酒或吸烟等 | ①有 次；　　②没有 |
| 8. 故意吞食异物（无法消化的物品） | ①有 次；　　②没有 |

**五、生活经历** 很多孩子可能在16岁之前受到父母或家里其他人的打、骂，或其他形式的对待。请你对以下所描述的情形，按照自己在过去时间的实际情况进行选择。

| **1. 曾经有人狠狠地掐你、拧你或重重地用指关节敲打你吗？** | | | | | |
| --- | --- | --- | --- | --- | --- |
| ①这种伤害行为发生过多少次？ | 没发生 | 偶尔 | 有时 | 经常 | 常常 |
| ②伤害你的人是谁？**（可多选）** | 没发生 | 父母 其他人 | |  | |
| ③这种行为对你的伤害达到什么程度？ | 没发生 | 没有伤害 | 轻度 | 中度 | 重度 |
| ④这种行为发生在什么时间? **(可多选）** | 没发生 | ≤12岁 | | 13岁以后 | |
| **2. 曾经有人狠狠地用手或拳头打你的头部和脸部（包括打耳光和拽头发）吗？** | | | | | |
| ①这种伤害行为发生过多少次？ | 没发生 | 偶尔 | 有时 | 经常 | 常常 |
| ②伤害你的人是谁？**（可多选）** | 没发生 | 父母 其他人 | |  | |
| ③这种行为对你的伤害达到什么程度？ | 没发生 | 没有伤害 | 轻度 | 中度 | 重度 |
| ④这种行为发生在什么时间? **(可多选）** | 没发生 | ≤12岁 | | 13岁以后 | |
| **3. 曾经人用手或拳头狠狠地打你身体（不包括头部和脸部）或用脚踢你吗？** | | | | | |
| ①这种伤害行为发生过多少次？ | 没发生 | 偶尔 | 有时 | 经常 | 常常 |
| ②伤害你的人是谁？**（可多选）** | 没发生 | 父母 其他人 | |  | |
| ③这种行为对你的伤害达到什么程度？ | 没发生 | 没有伤害 | 轻度 | 中度 | 重度 |
| ④这种行为发生在什么时间? **(可多选）** | 没发生 | ≤12岁 | | 13岁以后 | |
| **4. 曾经有人用皮带或用棍、棒、木板等硬东西打你吗？** | | | | | |
| ①这种伤害行为发生过多少次？ | 没发生 | 偶尔 | 有时 | 经常 | 常常 |
| ②伤害你的人是谁？**（可多选）** | 没发生 | 父母 其他人 | |  | |
| ③这种行为对你的伤害达到什么程度？ | 没发生 | 没有伤害 | 轻度 | 中度 | 重度 |
| ④这种行为发生在什么时间? **(可多选）** | 没发生 | ≤12岁 | | 13岁以后 | |
| **5. 曾经有人强迫你跪在地上或站在某个地方很长时间吗？** | | | | | |
| ①这种伤害行为发生过多少次？ | 没发生 | 偶尔 | 有时 | 经常 | 常常 |
| ②伤害你的人是谁？**（可多选）** | 没发生 | 父母 其他人 | |  | |
| ③这种行为对你的伤害达到什么程度？ | 没发生 | 没有伤害 | 轻度 | 中度 | 重度 |
| ④这种行为发生在什么时间? **(可多选）** | 没发生 | ≤12岁 | | 13岁以后 | |
| **6. 曾经有人当着别人的面训斥你或故意不理你吗？** | | | | | |
| ①这种伤害行为发生过多少次？ | 没发生 | 偶尔 | 有时 | 经常 | 常常 |
| ②伤害你的人是谁？**（可多选）** | 没发生 | 父母 其他人 | |  | |
| ③这种行为对你的伤害达到什么程度？ | 没发生 | 没有伤害 | 轻度 | 中度 | 重度 |
| ④这种行为发生在什么时间? **(可多选）** | 没发生 | ≤12岁 | | 13岁以后 | |
| **7. 曾经有成年人用言语恶毒地骂你、讽刺你或侮辱你吗？** | | | | | |
| ①这种伤害行为发生过多少次？ | 没发生 | 偶尔 | 有时 | 经常 | 常常 |
| ②伤害你的人是谁？**（可多选）** | 没发生 | 父母 其他人 | |  | |
| ③这种行为对你的伤害达到什么程度？ | 没发生 | 没有伤害 | 轻度 | 中度 | 重度 |
| ④这种行为发生在什么时间? **(可多选）** | 没发生 | ≤12岁 | | 13岁以后 | |
| **8. 曾经有成年人经恐吓你或威胁你（如威胁要抛弃你）吗？** | | | | | |
| ①这种伤害行为发生过多少次？ | 没发生 | 偶尔 | 有时 | 经常 | 常常 |
| ②伤害你的人是谁？**（可多选）** | 没发生 | 父母 其他人 | |  | |
| ③这种行为对你的伤害达到什么程度？ | 没发生 | 没有伤害 | 轻度 | 中度 | 重度 |
| ④这种行为发生在什么时间? **(可多选）** | 没发生 | ≤12岁 | | 13岁以后 | |
| **9. 曾经有成年人无故拒绝你的合理要求，甚至向你大发脾气吗？** | | | | | |
| ①这种伤害行为发生过多少次？ | 没发生 | 偶尔 | 有时 | 经常 | 常常 |
| ②伤害你的人是谁？**（可多选）** | 没发生 | 父母 其他人 | |  | |
| ③这种行为对你的伤害达到什么程度？ | 没发生 | 没有伤害 | 轻度 | 中度 | 重度 |
| ④这种行为发生在什么时间? **(可多选）** | 没发生 | ≤12岁 | | 13岁以后 | |
| **10. 曾经有人（成年人或年长你5岁）对你耍流氓或动手动脚吗？** | | | | | |
| ①这种伤害行为发生过多少次？ | 没发生 | 偶尔 | 有时 | 经常 | 常常 |
| ②伤害你的人是谁？**（可多选）** | 没发生 | 父母 其他人 | |  | |
| ③这种行为对你的伤害达到什么程度？ | 没发生 | 没有伤害 | 轻度 | 中度 | 重度 |
| ④这种行为发生在什么时间? **(可多选）** | 没发生 | ≤12岁 | | 13岁以后 | |
| **11. 曾经有人（成年人或年长你5岁）强迫与你发生性接触（如性交行为等）吗？** | | | | | |
| ①这种伤害行为发生过多少次？ | 没发生 | 偶尔 | 有时 | 经常 | 常常 |
| ②伤害你的人是谁？**（可多选）** | 没发生 | 父母 其他人 | |  | |
| ③这种行为对你的伤害达到什么程度？ | 没发生 | 没有伤害 | 轻度 | 中度 | 重度 |
| ④这种行为发生在什么时间? **(可多选）** | 没发生 | ≤12岁 | | 13岁以后 | |
| **12. 曾经有人（成年人或年长你5岁）在你面前故意暴露、或让你抚摸他/她的生殖器吗？** | | | | | |
| ①这种伤害行为发生过多少次？ | 没发生 | 偶尔 | 有时 | 经常 | 常常 |
| ②伤害你的人是谁？**（可多选）** | 没发生 | 父母 其他人 | |  | |
| ③这种行为对你的伤害达到什么程度？ | 没发生 | 没有伤害 | 轻度 | 中度 | 重度 |
| ④这种行为发生在什么时间? **(可多选）** | 没发生 | ≤12岁 | | 13岁以后 | |
| **13. 曾经有人（成年人或年长你5岁）让你看色情图片、录像，或给你讲下流的故事吗？** | | | | | |
| ①这种伤害行为发生过多少次？ | 没发生 | 偶尔 | 有时 | 经常 | 常常 |
| ②伤害你的人是谁？**（可多选）** | 没发生 | 父母 其他人 | |  | |
| ③这种行为对你的伤害达到什么程度？ | 没发生 | 没有伤害 | 轻度 | 中度 | 重度 |
| ④这种行为发生在什么时间? **(可多选）** | 没发生 | ≤12岁 | | 13岁以后 | |

**Questionnaire _ Senior middle school Version**

**One**

| **1. Gender** ① boy； ② girl |
| --- |
| **2. Grade** ① one； ② two； ③ three |
| **3. Birth date** |
| **4. Registered residence** ① urban； ② rural |
| **5. Are you a only child?**  ① yes； ② no |
| **6．How much education does your father have?** |
| ① less than junior middle school； ② junior middle school； |
| ③ senior middle school； ④ college or more |
| **7．How much education does your mother have?** |
| ① less than junior middle school； ② junior middle school； |
| ③ senior middle school； ④ college or more |
| **8．How do you think about your family economic status compared with other students?** |
| ① very poor； ② poor； ③ moderate； ④ good； ⑤ very good |
| **9．How many closed friends do you have?** |
| ① none； ② 1-2； ③ 3-5； ④ ≥6 |

**Two**

| **1. How many times had you seriously considered attempting suicide during the 12 months before**  **the survey?** |
| --- |
| 1. none； ② 1； ③ 2-3； ④ ≥4 |
| **2. How many times had you made a plan about how you would attempt suicide during the 12 months before the survey?**  ① none； ② 1； ③ 2-3； ④ ≥4 |
| **3. How many times had you attempted suicide during the 12 months before the survey?** |
| ① none； ② 1； ③ 2-3； ④ ≥4 |

**Three**

| **Questions** | | **lasting ≥3 months** | **lasting ≥2 months** | **lasting ≥1 month** | **lasting ≥2 weeks** | **lasting ≥1 week** | **none or lasting< 1 week** |
| --- | --- | --- | --- | --- | --- | --- | --- |
|  | Do you always feel indifference to anything? | **1** | **2** | **3** | **4** | **5** | **6** |
|  | Do you always feel difficult to remember things you’ve learned? | **1** | **2** | **3** | **4** | **5** | **6** |
|  | Do you always feel restless without reason? | **1** | **2** | **3** | **4** | **5** | **6** |
|  | Do you always feel other people are unfriendly to you? | **1** | **2** | **3** | **4** | **5** | **6** |
|  | Do you always feel anxiety without reason? | **1** | **2** | **3** | **4** | **5** | **6** |
|  | | | | | | | |
|  | Do you always feel that others can know your private thoughts? | **1** | **2** | **3** | **4** | **5** | **6** |
|  | Do you blame yourself frequently? | **1** | **2** | **3** | **4** | **5** | **6** |
|  | Do you often feel someone is talking about you? | **1** | **2** | **3** | **4** | **5** | **6** |
|  | Do you often feel fear about empty places? | **1** | **2** | **3** | **4** | **5** | **6** |
|  | Do you often feel difficult to study at home? | **1** | **2** | **3** | **4** | **5** | **6** |
|  | | | | | | | |
|  | Are you often hesitant to do things? | **1** | **2** | **3** | **4** | **5** | **6** |
|  | Do you always feel that most people can't be trusted? | **1** | **2** | **3** | **4** | **5** | **6** |
|  | Do you often feel afraid to go to public places? | **1** | **2** | **3** | **4** | **5** | **6** |
|  | Are you often unaccustomed to school life? | **1** | **2** | **3** | **4** | **5** | **6** |
|  | Do you often feel irritable without reason? | **1** | **2** | **3** | **4** | **5** | **6** |
|  | | | | | | | |
|  | Do you often reluctant to talk to other people when feeling down in the dumps? | **1** | **2** | **3** | **4** | **5** | **6** |
|  | Do you often feel afraid without reason? | **1** | **2** | **3** | **4** | **5** | **6** |
|  | Do you often feel unhopeful about your future? | **1** | **2** | **3** | **4** | **5** | **6** |
|  | Do you often feel difficult to concentrate on something? | **1** | **2** | **3** | **4** | **5** | **6** |
|  | Do you always feel hard to get used to the teacher's teaching method? | **1** | **2** | **3** | **4** | **5** | **6** |
|  | | | | | | | |
|  | Do you always feel distressed? | **1** | **2** | **3** | **4** | **5** | **6** |
|  | Do you always have the impulse to damage something? | **1** | **2** | **3** | **4** | **5** | **6** |
|  | Do you always have unnecessary thoughts? | **1** | **2** | **3** | **4** | **5** | **6** |
|  | Do you usually not want to ask for help when in trouble? | **1** | **2** | **3** | **4** | **5** | **6** |
|  | Do you often feel afraid of teacher’ question in class? | **1** | **2** | **3** | **4** | **5** | **6** |
|  |  |  |  |  |  |  |  |
|  | Do you usually not enjoy talking about problems with classmates? | **1** | **2** | **3** | **4** | **5** | **6** |
|  | Do you often think of death? | **1** | **2** | **3** | **4** | **5** | **6** |
|  | Do you often not work  up much enthusiasm for anything? | **1** | **2** | **3** | **4** | **5** | **6** |
|  | Do you often make a plan to attempt suicide? | **1** | **2** | **3** | **4** | **5** | **6** |
|  | Do you often argue with others and have to fight it out? | **1** | **2** | **3** | **4** | **5** | **6** |
|  | | | | | | | |
|  | Do you always feel strange in a large group of people? | **1** | **2** | **3** | **4** | **5** | **6** |
|  | Do you always feel that others are against you? | **1** | **2** | **3** | **4** | **5** | **6** |
|  | Do you always feel stressed when you are alone? | **1** | **2** | **3** | **4** | **5** | **6** |
|  | Do you always hate to go to school? | **1** | **2** | **3** | **4** | **5** | **6** |
|  | Are you always out of control in your terrible temper? | **1** | **2** | **3** | **4** | **5** | **6** |
|  | | | | | | | |
|  | Do you always feel fidgeting when the exam is coming? | **1** | **2** | **3** | **4** | **5** | **6** |
|  | Do you often feel angry with some minor issues? | **1** | **2** | **3** | **4** | **5** | **6** |
|  | Do you often find it’s hard to learn compared with classmates? | **1** | **2** | **3** | **4** | **5** | **6** |
|  | Do you often feel very nervous when being looked at? | **1** | **2** | **3** | **4** | **5** | **6** |

**Four**

***Within the last year, have you harmed yourself in a way that was deliberate but not***

***intended as a means by which to take your life?***

| **Methods** | Yes or No |
| --- | --- |
| 1. hit yourself? | ① Yes；　　② No |
| 2. pulled your own hair? | ① Yes；　　② No |
| 3. banged your head or fist against something? | ① Yes；　　② No |
| 4. pinched or scratched yourself? | ① Yes；　　② No |
| 5. bitten yourself? | ① Yes；　　② No |
|  |  |
| 6. cut or pierced yourself? | ① Yes；　　② No |
| 7. taken an overdose (e.g. of pills, alcohol or cigarette)? | ① Yes；　　② No |
| 8. ingested a non-ingestible substance or object? | ① Yes；　　② No |

**Five**

***While you were growing up (during your first*** ***16 years of life)…?”***

| ***1.* *Did a parent or other adult in the household ever push, grab, pinch, or throw something at you seriously?*** | | | | | |
| --- | --- | --- | --- | --- | --- |
| *① How often did someone do any of these things to you?* | No abuse | Occasionally | sometimes | often | very often |
| *② Who is the perpetrator？****（Multiple response）*** | No abuse | Parent | | Others | |
| *③ What’s your perceived harm of these things？* | No abuse | No harm | Mild | Moderate | Severe |
| *④* *When did these things happen?****（Multiple response）*** | No abuse | ≤12y | | 12y–16y | |
| ***2. Did a parent or other adult in the household ever slap you on the arm, hand, leg, head, ears, or face seriously?*** | | | | | |
| *① How often did someone do any of these things to you?* | No abuse | Occasionally | sometimes | often | very often |
| *② Who is the perpetrator？****（Multiple response）*** | No abuse | Parent | | Others | |
| *③ What’s your perceived harm of these things？* | No abuse | No harm | Mild | Moderate | Severe |
| *④* *When did these things happen?****（Multiple response）*** | No abuse | ≤12y | | 12y–16y | |
| ***3. Did a parent or other adult in the household ever hit you with fist or kick you hard?*** | | | | | |
| *① How often did someone do any of these things to you?* | No abuse | Occasionally | sometimes | often | very often |
| *② Who is the perpetrator？****（Multiple response）*** | No abuse | Parent | | Others | |
| *③ What’s your perceived harm of these things？* | No abuse | No harm | Mild | Moderate | Severe |
| *④* *When did these things happen?****（Multiple response）*** | No abuse | ≤12y | | 12y–16y | |
| ***4. Did a parent or other adult in the household ever hit you on some part of your body with something like a belt, hairbrush, stick, or some other hard object?*** | | | | | |
| *① How often did someone do any of these things to you?* | No abuse | Occasionally | sometimes | often | very often |
| *② Who is the perpetrator？****（Multiple response）*** | No abuse | Parent | | Others | |
| *③ What’s your perceived harm of these things？* | No abuse | No harm | Mild | Moderate | Severe |
| *④* *When did these things happen?（****Multiple response）*** | No abuse | ≤12y | | 12y–16y | |
| ***5. Did a parent or other adult in the household ever force you to kneel on the ground or stand for a long time?*** | | | | | |
| *① How often did someone do any of these things to you?* | No abuse | Occasionally | sometimes | often | very often |
| *② Who is the perpetrator？****（Multiple response）*** | No abuse | Parent | | Others | |
| *③ What’s your perceived harm of these things？* | No abuse | No harm | Mild | Moderate | Severe |
| *④* *When did these things happen?（****Multiple response）*** | No abuse | ≤12y | | 12y–16y | |
| ***6. Did a parent or other adult in the household ever shout, yell, or scream at you in front of others?*** | | | | | |
| *① How often did someone do any of these things to you?* | No abuse | Occasionally | sometimes | often | very often |
| *② Who is the perpetrator？****（Multiple response）*** | No abuse | Parent | | Others | |
| *③ What’s your perceived harm of these things？* | No abuse | No harm | Mild | Moderate | Severe |
| *④* *When did these things happen?（****Multiple response）*** | No abuse | ≤12y | | 12y–16y | |
| ***7. Did a parent or other adult in the household ever swear or curse at you?*** | | | | | |
| *① How often did someone do any of these things to you?* | No abuse | Occasionally | sometimes | often | very often |
| *② Who is the perpetrator？****（Multiple response）*** | No abuse | Parent | | Others | |
| *③ What’s your perceived harm of these things？* | No abuse | No harm | Mild | Moderate | Severe |
| *④* *When did these things happen?****（Multiple response）*** | No abuse | ≤12y | | 12y–16y | |
| ***8. Did a parent or other adult in the household ever send you away or kick you out of the house?*** | | | | | |
| *① How often did someone do any of these things to you?* | No abuse | Occasionally | sometimes | often | very often |
| *② Who is the perpetrator？****（Multiple response）*** | No abuse | Parent | | Others | |
| *③ What’s your perceived harm of these things？* | No abuse | No harm | Mild | Moderate | Severe |
| *④* *When did these things happen?****（Multiple response）*** | No abuse | ≤12y | | 12y–16y | |
| ***9. Did a parent or other adults in the household ever call you dumb, lazy, or another name like that?*** | | | | | |
| *① How often did someone do any of these things to you?* | No abuse | Occasionally | sometimes | often | very often |
| *② Who is the perpetrator？****（Multiple response）*** | No abuse | Parent | | Others | |
| *③ What’s your perceived harm of these things？* | No abuse | No harm | Mild | Moderate | Severe |
| *④* *When did these things happen?****（Multiple response）*** | No abuse | ≤12y | | 12y–16y | |
| ***10. Did an adult or person at least five years older than you ever touch or fondle you in a sexual way?*** | | | | | |
| *① How often did someone do any of these things to you?* | No abuse | Occasionally | sometimes | often | very often |
| *② Who is the perpetrator？****（Multiple response）*** | No abuse | Parent | | Others | |
| *③ What’s your perceived harm of these things？* | No abuse | No harm | Mild | Moderate | Severe |
| *④* *When did these things happen?****（Multiple response）*** | No abuse | ≤12y | | 12y–16y | |
| ***11. Did an adult or person at least five years older than you ever attempted (or actually had) intercourse with you?*** | | | | | |
| *① How often did someone do any of these things to you?* | No abuse | Occasionally | sometimes | often | very often |
| *② Who is the perpetrator？****（Multiple response）*** | No abuse | Parent | | Others | |
| *③ What’s your perceived harm of these things？* | No abuse | No harm | Mild | Moderate | Severe |
| *④* *When did these things happen?****（Multiple response）*** | No abuse | ≤12y | | 12y–16y | |
| ***12. Did an adult or person at least five years older than you ever have you touch their body in a sexual way?*** | | | | | |
| *① How often did someone do any of these things to you?* | No abuse | Occasionally | sometimes | often | very often |
| *② Who is the perpetrator？****（Multiple response）*** | No abuse | Parent | | Others | |
| *③ What’s your perceived harm of these things？* | No abuse | No harm | Mild | Moderate | Severe |
| *④* *When did these things happen?****（Multiple response）*** | No abuse | ≤12y | | 12y–16y | |
| ***13. Did an adult or person at least five years older than you ever force you to watch pornographic pictures or videos or talk dirty to you?*** | | | | | |
| *① How often did someone do any of these things to you?* | No abuse | Occasionally | sometimes | often | very often |
| *② Who is the perpetrator？****（Multiple response）*** | No abuse | Parent | | Others | |
| *③ What’s your perceived harm of these things？* | No abuse | No harm | Mild | Moderate | Severe |
| *④* *When did these things happen?****（Multiple response）*** | No abuse | ≤12y | | 12y–16y | |
